# Unraveling the Developmental Roadmap toward Human Brown AdiposeTissue

**DOI:** 10.1016/j.stemcr.2021.03.009

**Published:** 2021-04-13

**Authors:** Stefania Carobbio, Anne-Claire Guenantin, Myriam Bahri, Sonia Rodriguez-Fdez, Floris Honig, Ioannis Kamzolas, Isabella Samuelson, Kathleen Long, Sherine Awad, Dunja Lukovic, Slaven Erceg, Andrew Bassett, Sasha Mendjan, Ludovic Vallier, Barry S. Rosen, Davide Chiarugi, Antonio Vidal-Puig

## Main text

(Stem Cell Reports *16*, 641–655; March 9, 2021)

The authors have amended the GEO number provided in the original article because it was not linked to the RNAseq datasets used in the study. The correct GEO number is GSE168387. This now appears in the manuscript online.

